# TRIM28-Mediated Excessive Oxidative Stress Induces Cellular Senescence in Granulosa Cells and Contributes to Premature Ovarian Insufficiency In Vitro and In Vivo

**DOI:** 10.3390/antiox13030308

**Published:** 2024-03-01

**Authors:** Chong Zhou, Dandan Li, Jinxia He, Tao Luo, Yiting Liu, Yue Xue, Jian Huang, Liping Zheng, Jia Li

**Affiliations:** 1 School of Basic Medical Sciences, Jiangxi Medical College, Nanchang University, Nanchang 330031, China; 356400210004@email.ncu.edu.cn (C.Z.); luotao@ncu.edu.cn (T.L.); 406400230104@email.ncu.edu.cn (Y.L.); 356400220010@email.ncu.edu.cn (Y.X.); 2 Key Laboratory of Reproductive Physiology and Pathology of Jiangxi Province, Jiangxi Medical College, Nanchang University, Nanchang 330031, China; 4202120020@email.ncu.edu.cn (D.L.); jianhuang@ncu.edu.cn (J.H.); 3 HuanKui College, Nanchang University, Nanchang 330031, China; 4 Reproductive Medical Center, Jiangxi Maternal and Child Health Hospital, Affiliated Maternal and Child Health Hospital of Nanchang University, Nanchang 330006, China; 401428818004@email.ncu.edu.cn; 5 Institute of Life Science, Nanchang University, Nanchang 330031, China; 6 School of Public Health, Jiangxi Medical College, Nanchang University, Nanchang 330006, China; 7 Jiangxi Provincial Key Laboratory of Preventive Medicine, Jiangxi Medical College, Nanchang University, Nanchang 330006, China

**Keywords:** premature ovarian insufficiency, granulosa cells, cellular senescence, oxidative stress, autophagy, TRIM28

## Abstract

Premature ovarian insufficiency (POI) is a clinical syndrome of ovarian dysfunction characterized by the abnormal alteration of hormone levels such as FSH and E_2_. POI causes infertility, severe daily life disturbances, and long-term health risks. However, the underlying mechanism remains largely unknown. In this study, we found that POI is associated with the cellular senescence of ovarian granulosa cells, and TRIM28 mediates oxidative stress (OS)-induced cellular senescence in granulosa cells. Mechanistically, OS causes a decrease in TRIM28 protein levels in KGN cells. Subsequently, it triggers an increase in the levels of autophagy marker proteins ATG5 and LC3B-II, and the downregulation of P62. Abnormal autophagy induces an increase in the levels of cellular senescence markers γ-H2A.X, P16, and P21, provoking cellular senescence in vitro. The overexpression of ovarian TRIM28 through a microinjection of lentivirus attenuated autophagy, cellular senescence, and follicular atresia in the ovaries of POI mice and improved mouse fertility in vivo. Our study highlights the triggers for POI, where the reduction of TRIM28, which is regulated by reactive oxygen species, causes follicular atresia and POI via triggering autophagy and inducing granulosa cell senescence. Shedding light on TRIM28 may represent a potential intervention strategy for POI.

## 1. Introduction

Premature ovarian insufficiency (POI) poses a significant threat to women’s reproductive health. It is classically defined as the absence of menstruation for a period of 4–6 months in women under the age of 40, accompanied by elevated follicle-stimulating hormone (FSH) and low estradiol levels [1]. Therefore, understanding the pathogenesis of POI plays a crucial role in its clinical diagnosis and treatment. Currently, POI is classified into different subtypes based on its progression, namely, occult, biochemical, and overt types [1,2]. In this study, we focused on biochemical premature ovarian insufficiency (bPOI) as patients in the bPOI stage have a greater potential for the restoration of ovarian function, and their associated hormone levels are altered sufficiently to be observed. However, an increase in atretic follicles and a decrease in the number of follicles is a distinctive feature observed regardless of the stage of POI. As the fundamental unit of female reproduction, the ovarian follicle consists of an oocyte surrounded by granulosa and theca cells. Importantly, abnormalities in granulosa cells (GCs) can lead to follicular atresia [3], resulting in a reduction in the number of follicles within the ovarian reserve pool and ultimately causing POI, but it remains unclear how the granulosa cells influence the development of POI.

The excessive production of reactive oxygen species (ROS) and impaired antioxidant defense mechanisms lead to oxidative stress (OS) [4,5], which is considered to be the main cause of follicular atresia [6,7]. Numerous studies have reported significantly higher levels of oxidative stress in the POI group compared to the control group, suggesting that OS can act as a biomarker of POI [8,9,10]. As the main source of intracellular ROS, mitochondria play a very critical role in cellular redox homeostasis [11]. However, mitochondria are also a major target of ROS oxidative attack. When ROS generation exceeds the scavenging capacity of antioxidant defenses, excess ROS can disrupt mitochondrial integrity, inhibit the activity of the electron transport chain enzyme complex, lead to the collapse of the mitochondrial membrane potential (MMP) [12], and cause mitochondrial dysfunction, which ultimately increases ROS production and exacerbates cellular damage such as cellular senescence [13], autophagy [14], and apoptosis [15].

Cellular senescence is a state of irreversible growth arrest induced by various cellular stresses such as telomere dysfunction, DNA damage, OS, and oncogenic mutations [16]. The free radical theory of aging suggests that the accumulation of macromolecular damage by ROS is one of the molecular mechanisms of cellular senescence [17]. Meanwhile, there is compelling evidence that cellular senescence is associated with the development of diseases such as endometriosis-associated infertility and cardiac senescence [18,19]. However, the role of OS-induced cellular senescence in the development of POI is still unclear.

Apoptosis and autophagy in GCs have been shown to be due to excessive ROS production from OS [20,21]. ROS generated in vivo and in vitro accelerate apoptosis in follicular GCs [22,23]. In addition, oxidative damage to GCs during follicular atresia can induce autophagy [24]. Recent studies have shown that autophagy is associated with cellular responses to OS. Under normal physiological conditions, mammalian cells usually exhibit low levels of autophagic activity in response to ROS, whereas excessive autophagy triggers cellular senescence and even death in response to OS [25]. The mechanism of occurrence between OS-induced autophagy and ovarian aging needs to be elucidated in further studies.

The tripartite motif-containing protein superfamily (TRIMs) are involved in diverse biological processes and play a crucial role in cancer therapy [26,27,28,29,30]. Recent evidence suggests that TRIM28 regulates the post-translational modification of proteins and participates in autophagy [31,32,33]. However, the involvement of TRIM28 in the regulation of autophagy in GCs of POI remains to be explored. Therefore, it is of great significance to investigate the role of TRIM28-mediated autophagy in GC senescence, which is expected to reveal new targets for the treatment of POI.

In this study, we explored the pathogenesis of POI, in which TRIM28-mediated OS induces senescence lesions of GCs. We show that the accumulation of ROS induces mitochondrial dysfunction, which triggers intracellular OS, at which time the expression of the autophagy-related protein TRIM28 is inhibited, leading to alterations in the protein levels of the key autophagy participants including autophagy-related gene 5 (ATG5, involved in the assembly of autophagy precursors [34]), sequestosome 1 (P62, autophagy junction protein [35], which is selectively wrapped into autophagosomes), and microtubule-associated protein 1 light chain 3b (LC3B, involved in the formation of autophagosomes; during autophagy, LC3B-I in the cytosol is processed by ATG7 and ATG3, which couples with phosphatidylethanolamine to form LC3B-II and localize to autophagosomes [36]). This, ultimately, results in altered protein levels of the senescence marker proteins including cyclin-dependent kinase inhibitor 1A and cyclin-dependent kinase inhibitor 2A (P21 and P16, which mediate cell cycle arrest, indicative of cellular senescence [37]) and phorspho-histone H2A.X (γ-H2A.X, which occurs as a result of DNA damage, which is an important feature of senescence [38]). In vivo, the overexpression of TRIM28 in the ovary restores ovarian function, including follicular atresia and hormone levels that reflect the GCs status in POI mice, while levels of the OS-related proteins heme Oxygenase-1 and superoxide Dismutase 2 (HO-1 and SOD2, antioxidant enzymes that reflect levels of oxidative stress [39]) and autophagy are decreased in the ovary. Unraveling the mechanisms involved in GC senescence and elucidating the pathological changes of GCs in POI may contribute to the development of new strategies to improve ovarian function and pregnancy outcomes.

## 2. Material and Methods

### 2.1. Study Approval

The study was approved and monitored by the Ethics Committee of the Second Affiliated Hospital of Nanchang University ((2023) CDEFYYLK (03-032)). Informed written consent was obtained from each patient before sample collection. Female or male KM mice were maintained in accordance with the National Institutes of Health Guide for the Care and Use of Laboratory Animals, and the experiments were approved by the Committee of Experimental Animal Ethics, Nanchang University (NCU2022101401).

### 2.2. Collection of Human Follicular Fluids

Human follicular fluids (FFs) were obtained from patients with POI (*n* = 15) and healthy women (CTRL, *n* = 15); they had undergone IVF treatment in the Reproductive Medical Center of the Second Affiliated Hospital of Nanchang University (Nanchang, Jiangxi, China) between March 2022 and May 2023. This study was approved by the ethics committee of Jiangxi Provincial Maternal and Child Health Hospital. Written informed consent was signed by the participants or their relatives. Subjects were classified into the POI and control groups according to the Bologna criteria and the European Society of Human Reproduction and Embryology (ESHRE) guidelines [40]. The process involved a comprehensive consideration of the various definitions of POI [41] to generate the standard set of POI formulations.

### 2.3. Animal Model

Female Kunming mice (KM) as a model for the age-related decline of female fertility in humans [42] were housed in specific pathogen-free conditions at the Nanchang University Experimental Animal Center, and were fed cobalt-60-irradiated and SPF-grade chow. For the induction of the mouse POI model, the 6-week-old female KM mice were intraperitoneally administered 70 mg/kg cytoxan (CTX) (C0768, Sigma-Aldrich, St. Louis, MO, USA) and 12 mg/kg busulfan (BU) (B2635, Sigma-Aldrich, St. Louis, MO, USA) once a week for two weeks according to previous studies [43]. The control group was injected with an equal volume of normal saline. All mice were maintained at a constant temperature of 22 ± 2 °C and a relative humidity of 55 ± 10%. The mice were housed in plastic cages (4 mice in a cage) with a 12:12 h light/dark cycle throughout the study.

### 2.4. Culture of Primary Human Granulosa Cells (hGCs)

The hGCs were obtained from FFs collected during oocyte extraction in women undergoing IVF, as previously described [44]. Briefly, FFs were centrifuged at 400× *g* for 5 min, supernatants were removed, and cells were resuspended with 4 mL of DMEM/F12 medium; then, they were added to 8 mL of Ficoll Plus (P4350, Solarbio, Beijing, China) and centrifuged at 400× *g* for 15 min at room temperature to isolate the hGCs. Purified hGCs were inoculated into 12-well plates and cultured in DMEM/F12 medium supplemented with 10% fetal bovine serum (FBS; HyClone Laboratories, Lohan, UT, USA), 1 × GlutaMAX (Thermo Fisher Scientific/Invitrogen, Waltham, MA, USA), 100 mg/mL streptomycin sulfate (Thermo Fisher Scientific/Invitrogen, Waltham, MA, USA), and 100 U/mL penicillin (Thermo Fisher Scientific/Invitrogen, Waltham, MA, USA) at an incubation temperature of 37 °C and a humidity of 5% CO_2_. The medium was changed the next day and every other day for up to 5 days.

### 2.5. Cell Lines and Treatments

The human GC line KGN was purchased from Procell (CL-0603, Wuhan, China). H_2_O_2_ at 100 μM was used to induce OS. Cells were treated with or without 100 μM H_2_O_2_ for 24 h or pretreated with 5 mM NAC (HY-B0215, Med Chem Express, Monmouth Junction, NJ, USA) for 12 h, followed by 100 μM H_2_O_2_ for 24 h.

Cells were treated with 20 μM chloroquine (CQ) (M8699, Sigma, St. Louis, MO, USA) for 12 h before 100 μM H_2_O_2_ treatment for 24 h. Cells were transfected with Lenti-TRIM28 (pcSLenti-EF1-EGFP-P2A-Puro-CMV-TRIM28-3Xflag-WPRE, Oobic, Shanghai, China) according to the manufacturer’s protocol and were harvested 48 h post transfection for further analysis.

To knock down TRIM28, the KGN cells were cultured in vitro and transfected with TRIM28 siRNA (genOFFTM st-h- TRIM28 #1: 5′-GGACAAACATGCAACATTGCA-3′, #2: 5′-GGACTACAACCTTATTGTTAT-3′) or siRNA-negative control (NC, 5′-GTTCTCCG AACGTGTCACGT-3′) for 48 h according to the manufacturer’s instructions (SIGS0016288-1, Ribobio, Guangzhou, China). The day before transfection, 2 × 10^5^ KGN cells were seeded in 6-well plates at 60–70% confluence. The complete culture medium was replaced with culture medium containing siRNA-negative or siTRIM28. After 12 h, the siRNA was replaced with complete culture medium and the KGN cells were cultured for 48 h.

### 2.6. Western Blot Analysis

Total protein was extracted from GCs in human FFs, mouse ovarian tissue, and KGN cells using radioimmunoprecipitation assay (RIPA) buffer (R0010, Solarbio, Beijing, China) containing 1 × protease inhibitor cocktail (HY-K0010, Med Chem Express, Monmouth Junction, NJ, USA). Total protein (30 μg) was separated using sodium dodecyl sulfate-polyacrylamide gel electrophoresis (using a 12% gel) and transferred onto polyvinylidene difluoride membranes (IPVH00010, Sigma-Aldrich, St. Louis, MO, USA). Then, the membranes were incubated with the appropriate primary antibodies: anti-TRIM28 (1:1000, bs-17741R, Bioss, Beijing, China), anti-LC3B (1:1000, L7543, Sigma-Aldrich, St. Louis, MO, USA), anti-γ-H2AX (1:2000, 50599-2-Ig, Proteintech, Wuhan, China), anti-ATG5 (1:2000, 10181-2-AP, Proteintech, Wuhan, China), anti-P62 (1:2000, 10181-2-AP, Proteintech, Wuhan, China), anti-P16 (1:1000, 9664, Cell Signaling Technology, Danvers, MA, USA), P21 (1:1000, 10355-1-AP, Proteintech, Wuhan, China), HO-1 (1:1000, 10701-1-AP, Proteintech, Wuhan, China), and anti-Actin (1:5000, 10494-1-AP, Proteintech, Wuhan, China) for 14 h at 4 °C. Next, they were incubated with horseradish peroxidase (HRP)-conjugated goat anti-rabbit IgG (1:5000, SA00001-2, Proteintech, Wuhan, China) or goat anti-mouse IgG (1:5000, SA00001-1, Proteintech, Wuhan, China). The protein bands were visualized by using the enhanced chemiluminescence detection kit (S6009M, Uelandy, Shanghai, China). Analysis of the relative level of the target proteins was conducted by quantifying the gray value of target bands detected by the corresponding antibodies normalized to those detected by the anti-GAPDH antibody (1:10,000, 60004-1-Ig, Proteintech, Wuhan, China) using Image J software (version 1.44, National Institutes of Health, Bethesda, MD, USA). Each experiment was repeated at least three times.

### 2.7. Microinjection of Lentivirus In Vivo

All POI and control mice were treated without food but with freely available water for 12 h prior to the experiment. Mice were anesthetized with 1% sodium pentobarbital via intraperitoneal injection and then fixed in a prone position. A 1.0 cm longitudinal incision was cut at the side of the spine through the subcutaneous tissue, muscular layer, and peritoneum, through which both ovaries were removed. The TRIM28 overexpression lentivirus (3.5 × 10^8^ TU/mL) was injected into the POI ovarian envelope under the microscope in vivo; in the Mock group, lentivirus was replaced with the same volume of lentiviral empty vector. After injections, the ovaries were placed back into the abdomen, and incisions were disinfected and sutured. Those mice were fed freely for 1 week for environmental acclimation.

### 2.8. Definition of the Phase of the Estrous Cycle and the Fertility Rate

To determine the phase of the estrous cycle, the vaginal smears of mice in the three groups were taken at about 9 am every day for 11 consecutive days. The stage of the estrous cycles is judged by cytology under microscope [45], and the estrous cycle is divided into proestrus, estrus, and metestrus. Smears were taken continuously for 11 days. Meanwhile, the ovary was stained with hematoxylin and eosin (H&E), and a macroscopic and microscopic analysis of the ovaries was performed, and these were categorized as being in the follicular phase.

A mating test was carried out after 1 month of exposure. Selected sexually mature mice (male mice generally 2–3 months, female mice generally 3 months), two female mice and one male mouse of the same age, were kept in a cage, and the male mice were moved away after 10 days. The cage scheme was a control group female and wild-type male mouse, a POI group female and wild-type male mouse, and a TRIM28-OE group female and wild-type male mouse. The fertility situation was observed continuously, and the time and number of each fetus were recorded for about 6 months.

### 2.9. The Index of the Ovary

The mice were sacrificed, and the ovaries were removed under aseptic conditions and cleaned in phosphate-buffered saline (PBS) solution. The ovaries were weighed after removing the surface fluid with absorbent paper. The ovary index was calculated using the following formula: Ovary index (‰) = ovary weight/body weight × 1000 [46].

### 2.10. Histological Analysis of Ovarian Tissue and Ovarian Follicle Counts

We used the ovaries of mice fixed in a 4% paraformaldehyde solution; the ovaries were dehydrated in ethanol and xylene and embedded in paraffin. The paraffin-embedded ovaries were serially sectioned at 5 µm thickness by a microtome (RM2255, Leica, Berlin, Germany). Then, the ovarian sections were dewaxed in xylene, rehydrated in ethanol, and stained using H&E [47]. Then, all sections were viewed with a microscope (IX70, Olympus, Tokyo, Japan). The ovarian follicles were counted as described previously [48].

### 2.11. Measurement of Hormone Levels

Blood samples of mice were collected from the retroorbital plexus, and serum was collected to assess the levels of FSH (E-EL-M0511c, Elabscience, Wuhan, China), estradiol (E_2_) (E-EL-0150c, Elabscience, Wuhan, China), and anti-Müllerian hormone (AMH) (E-EL-M3015, Elabscience, Wuhan, China) by using the corresponding enzyme-linked immunosorbent assay (ELISA) according to the manufacturer’s instructions.

### 2.12. Autophagy Detection

CYTO-ID autophagy detection was performed using a CYTO-ID Autophagy Detection Kit 2.0 (ENZ-KIT175-0200, Enzo Life Science, Farmingdale, NY, USA). Briefly, THP-1 macrophages were stained with CYTO-ID Green dye (1:500) and Hoechst (1:1000) at 37 °C for 30 min. CYTO-ID Green dye is a cationic amphiphilic tracer dye that specifically labels autophagic compartments, indicating that spots stained with CYTO-ID are specific autophagic markers [49].

### 2.13. Detection of Cellular ATP Levels

ATP levels in GC lysates were measured using a luminometer (Synergy H4, BioTek, Winooski, VT, USA) according to the manufacturer’s instructions (S0027, Beyotime Biotechnology, Shanghai, China). Total protein was extracted from GC samples for normalization before the ATP assay.

### 2.14. Senescence-Associated β-Galactosidase (SA β-Gal) Assay

The SA β-Gal assay was carried out according to the manufacturer’s instructions (C0602, Beyotime Biotechnology, Shanghai, China). Freshly collected GCs were seeded on 12-well plates at a density of 5 × 10^5^ cells/mL. After 24 h, cells were washed, fixed, and stained in an X-gal solution overnight at 37 °C. Cells were imaged and photographed using a microscope (IX70, Olympus, Tokyo, Japan).

### 2.15. Immunofluorescence (IF)

The ovarian sections were incubated with 0.1% Triton X-100 for 1 h at room temperature. After blocking with 5% normal FBS for 1 h, the samples were incubated with the primary antibodies overnight at 4 °C. The primary antibodies were anti-TRIM28 (1:100, bs-17741R, Bioss, Beijing, China), LC3B (1:100, L7543, Sigma-Aldrich, St. Louis, MO, USA), and HO-1 (1:50, 10701-1-AP, Proteintech, Wuhan, China). Then, the samples were incubated with the secondary antibody (fluorescein isothiocyanate-conjugated goat anti-rabbit IgG, 1:100, E-AB-1014, Elabscience, Wuhan, China) for 1 h in the dark at room temperature.

### 2.16. Mitochondrial Membrane Potential Assay

Rhodamine (Rh) 123 staining was carried out according to the manufacturer’s instructions (C2008S, Beyotime Biotechnology, Shanghai, China). Freshly collected GCs were seeded on 12-well plates at a density of 5 × 10^5^ cells/mL. After 24 h, cells were washed, fixed, and stained in Rh123 solution for 30 min at 37 °C. Cells were imaged and photographed using a microscope (IX70, Olympus, Tokyo, Japan).

### 2.17. Statistical Methods

All analyses were performed using GraphPad Prism 5.01 (GraphPad Software, Inc., San Diego, CA, USA). The data are expressed as the mean ± standard error of the mean (SEM), and all data were analyzed by a normality test and ANOVA before comparison. Analysis of independent sample *t*-tests was used for the comparison between treated and control groups, and there was a statistically significant difference when *p* < 0.05 was applied (*p* * < 0.05 was considered significant; *p* ** < 0.01 and *p* *** < 0.001 were considered extremely significant).

## 3. Results

### 3.1. Granulosa Cells from bPOI Patients Show a Senescent Phenotype

**Figure 1:**
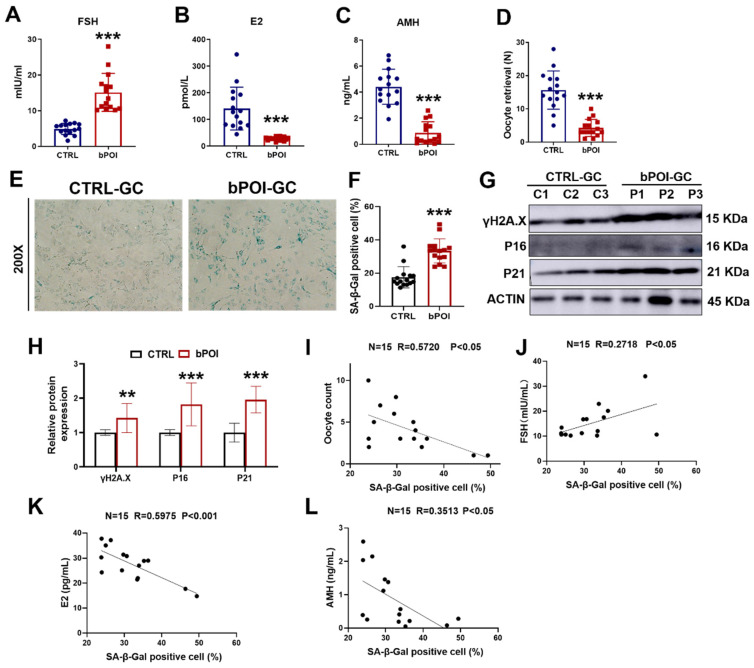
GCs from patients with bPOI show excessive OS and cellular senescence. (**A**–**C**) ELISA of FSH (**A**), E_2_ (**B**), and AMH (**C**) in human blood (*n* = 15 for controls, *n* = 15 for bPOI patients). (**D**) The number of oocytes of healthy women (*n* = 15) and bPOI patients (*n* = 15). (**E**) SA β-Gal assay of control-hGCs and bPOI-GCs (*n* = 15 for control-hGCs, *n* = 15 for bPOI-GCs, magnification, 200×). (**F**) The quantitative analysis of SA β-Gal positive cell (*n* = 15 for control-hGCs, *n* = 15 for bPOI-GCs). (**G**,**H**) Western blot of indicated proteins in GCs from control and POI patients (*n* = 15). GC, granulosa cell; C, control group; P, bPOI group. (**I**) Scatter diagram showing linear regression and significant Pearson correlation between the number of oocytes retrieved and SA β-Gal activity in bPOI-GCs based on the quantitative results of the SA β-Gal assay (*n* = 15). (**J**–**L**) Scatter diagram showing linear regression and significant Pearson correlation between SA β-Gal activity and the level of FSH (**J**), E_2_ (**K**), and AMH (**L**) in bPOI-GCs based on the quantitative results of the SA β-Gal assay (*n* = 15). ** *p* < 0.01, *** *p* < 0.001.

Clinical information was obtained from 15 healthy women and 15 bPOI in vitro fertilization (IVF) patients (Supplementary Table S1). Serum levels of follicle stimulating hormone (FSH) were significantly higher and levels of estrogen (E_2_) and anti-Müllerian hormone (AMH) were significantly lower in the bPOI patients compared to the control group (Figure 1A–C). We found that the number of oocytes retrieved was significantly reduced in patients with bPOI compared with the control group (Figure 1D). Since hormonal abnormalities characterize bPOI, and the formation and regulation of these hormones are closely related to granulosa cells (GCs), we isolated and extracted granulosa cells from clinical follicular fluid (FF) samples of the above 30 women. The cellular senescence of GCs was detected using senescence-associated β-galactosidase (SA β-Gal) staining, and we observed that the activity of SA β-Gal was increased in the GCs of bPOI patients compared with the control group (Figure 1E,F). Western blot showed that the protein levels of senescence-associated markers, γ-H2AX, P16, and P21, were elevated in bPOI-GCs (Figure 1G,H), indicating abnormal senescence in bPOI-GCs. Through linear regression analysis, we found that SA β-Gal activity in the GCs of bPOI patients was positively correlated with FSH (Figure 1J), and negatively correlated with the number of oocytes retrieved, E_2,_ and AMH content (Figure 1I,K,L). These results suggest that the occurrence of bPOI is associated with the abnormal senescence of GCs.

### 3.2. OS Accelerates Cellular Senescence in Granulosa Cells

**Figure 2:**
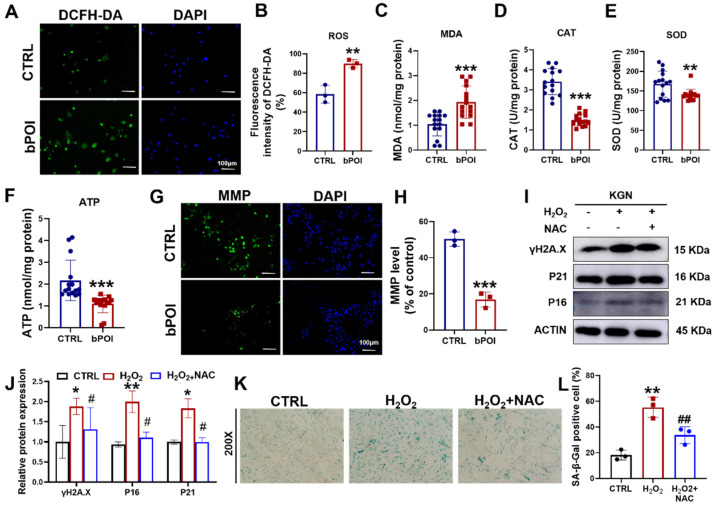
H_2_O_2_ induces mitochondrial dysfunction and cellular senescence in GCs cells. (**A**) The ROS levels assay in control-hGCs and bPOI-GCs using DCFH-DA fluorescent probes, scale bar: 100 μm. (**B**) The quantitative analysis of ROS levels (*n* = 3). (**C**–**F**) Intracellular MDA (**C**), CAT (**D**), SOD (**E**), and ATP (**F**) levels in GCs (*n* = 15 for control-hGCs, *n* = 15 for bPOI-GCs). (**G**) The level of MMP in control-hGCs and bPOI-GCs, scale bar: 100 μm. (**H**) The quantitative analysis of MMP level (*n* = 3). (**I**,**J**) The KGN cells were exposed to 100 μM H_2_O_2_ for 24 h or pretreated with 5 mM NAC, and the phosphorylation and total protein level of H2A.X, P21, and P16 were analyzed via Western blot. (**K**,**L**) The KGN cells were exposed to 100 μM H_2_O_2_ for 24 h or pretreated with 5 mM NAC, and the intracellular SA β-Gal levels were measured with a SA β-Gal staining kit (*n* = 3), magnification, 200×. ** *p* < 0.01, *** *p* < 0.001, compared with the control-hGCs, Student’s *t*-test. * *p* < 0.05, ** *p* < 0.01, compared with the control KGN cells; ^#^ *p* < 0.05, ^##^ *p* < 0.01, compared with the H_2_O_2_-treated group, one-way ANOVA.

OS is an important mechanism to induce cellular senescence [19]. We examined ROS levels in bPOI-GCs using DCFH-DA fluorescent probes. Compared to normal GCs, ROS levels were significantly elevated in bPOI-GCs (Figure 2A,B), and there was an increase in the content of the lipid peroxidation end product malondialdehyde (MDA) and a decrease in the activities of catalase (CAT) and superoxide dismutase (SOD) (Figure 2C–E). OS-induced cellular senescence is usually accompanied by mitochondrial dysfunction manifested in mitochondrial membrane potential imbalance and energy crisis [18]. We found that adenosine 5′-triphosphate (ATP) levels were decreased in bPOI-GCs (Figure 2F), and Rhodamine 123 staining revealed a significant decrease in mitochondrial membrane potential in bPOI-GCs (Figure 2G,H). This suggests that OS and further mitochondrial dysfunction occur in bPOI-GCs. Subsequently, to clarify whether OS is involved in the process of GC senescence, the GC line KGN was treated with H_2_O_2_, and the antioxidant N-Acetylcysteine (NAC) was added to observe the senescence of KGN cells. Western blot showed that protein levels of γ-H2AX, P16, and P21 were increased in H_2_O_2_-treated KGN cells, and NAC inhibited H_2_O_2_-induced protein levels of γ-H2AX, P16, and P21 in KGN cells (Figure 2I,J). The results showed that the activity of SA β-Gal was elevated in H_2_O_2_-treated KGN cells and that NAC inhibited the H_2_O_2_-induced SA β-Gal activity in KGN cells (Figure 2K,L).

### 3.3. HGCs from bPOI Patients Show Excessive Autophagy

**Figure 3:**
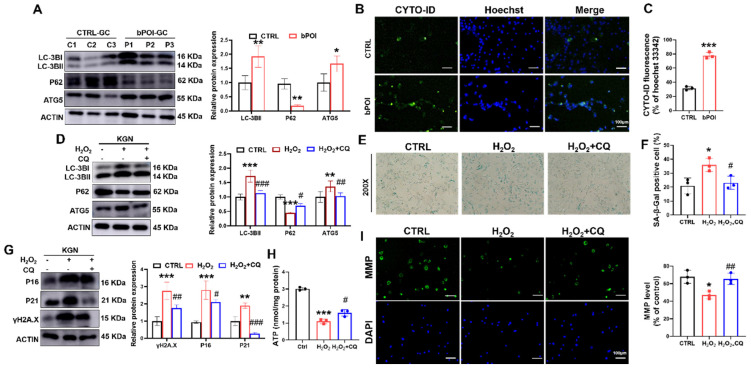
CQ attenuates OS-induced mitochondrial dysfunction and cellular senescence in KGN cells. (**A**) Western blot of LC3B, ATG5, and P62 in control-hGCs and bPOI-GCs (*n* = 3). (**B**,**C**) Fluorescent staining of intracellular autophagy levels using CYTO-ID Green dye in control and bPOI-GCs (*n* = 3), scale bar: 100 μm. (**D**) The KGN cells were exposed to 100 μM H_2_O_2_ for 24 h or pretreated with 20 μM CQ, and the protein levels of LC3B, P62, and ATG5 were analyzed via Western blot. (**E**,**F**) The KGN cells were exposed to 100 μM H_2_O_2_ for 24 h or pretreated with 20 μM CQ, and the intracellular SA β-Gal levels were measured with a SA β-Gal staining kit (*n* = 3), magnification, 200×. (**G**) The KGN cells were exposed to 100 μM H_2_O_2_ for 24 h or pretreated with 20 μM CQ, and the phosphorylation and total protein level of H2A.X, P21 and P16 were analyzed via Western blot. (**H**) Intracellular ATP levels in KGN cells treated with H_2_O_2_ or H_2_O_2_+CQ (*n* = 3). (**I**) The MMP level in KGN cells treated with H_2_O_2_ or H_2_O_2_+CQ (*n* = 3), scale bar: 100 μm. * *p* < 0.05, ** *p* < 0.01, *** *p* < 0.001, compared with the control-hGCs, Student’s *t*-test. * *p* < 0.05, ** *p* < 0.01, *** *p* < 0.001, compared with the control KGN cells; ^#^ *p* < 0.05, ^##^ *p* < 0.01, ^###^ *p* < 0.001, compared with the H_2_O_2_-treated group, one-way ANOVA.

Excessive autophagy results in the upregulation of autophagy-related gene 5 (ATG5) and LC3B-II and the downregulation of P62 [29]. The Western blot showed that the LC3B-II and ATG5 were significantly increased in bPOI-GCs compared with control-hGCs, and P62 protein level was significantly decreased compared with the control group (Figure 3A). The CYTO-ID probe binding to autophagic vesicles can release green fluorescence, which can be detected at the excitation wavelength of 488 nm. We detected a significant increase in the proportion of green fluorescence in the GCs of bPOI patients, suggesting that autophagy was increased in bPOI-GCs (Figure 3B,C).

### 3.4. Suppression of Autophagy Attenuates OS-Induced Senescence-Related Phenotypes in Granulosa Cells

To clarify whether autophagy is involved in OS-induced cellular senescence, we treated KGN cells with H_2_O_2_ to cause intracellular OS. Western blot revealed increased LC3B-II and ATG5 protein levels, and P62 protein level was down-regulated in H_2_O_2_-treated GCs (Figure 3D). And we found that chloroquine (CQ), a small molecule inhibitor of autophagy, reversed the H_2_O_2_-induced alterations in autophagy-associated protein levels (Figure 3D).

In addition, CQ also reduced the H_2_O_2_-induced increase in SA β-Gal activity and the increased protein levels of senescence markers P16, P21, and γ-H2AX in KGN cells, suggesting that the inhibition of autophagy was effective in slowing down OS-induced cellular senescence in KGN cells (Figure 3E–G). Further, we found that while H_2_O_2_ treatment decreased ATP levels, pretreatment with CQ for 12 h partially repaired ATP levels (Figure 3H). H_2_O_2_ treatment decreased the relative ratio of mitochondrial membrane potential (MMP) compared with control, but CQ significantly increased the ratio of mitochondrial membrane potential (MMP) compared with the H_2_O_2_-treated group (Figure 3I). These results suggest that the ROS-induced senescence of GCs is an autophagy-dependent mechanism.

### 3.5. Knockdown of TRIM28 Promotes Autophagy and Senescence-Related Phenotypes in GCs

**Figure 4:**
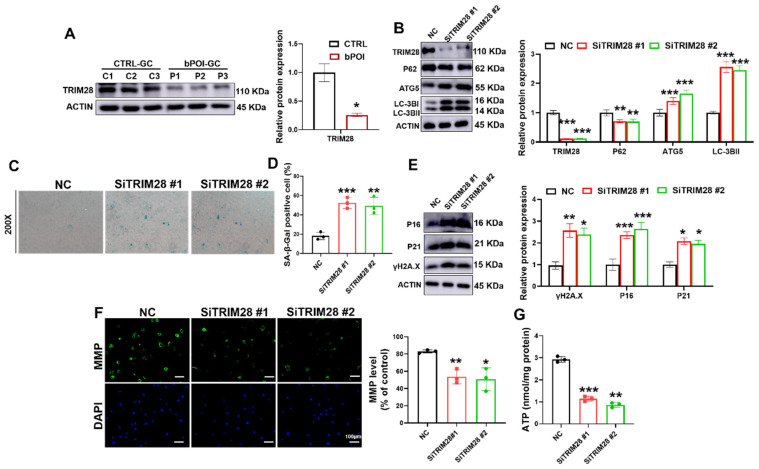
TRIM28 knockdown aggravates OS-induced senescence by promoting autophagy in vitro. (**A**) Western blot of TRIM28 in control-hGCs and bPOI-GCs (*n* = 3). (**B**) Western blot of LC3B, ATG5, and P62 in KGN cells transfected with siTRIM28. (**C**,**D**) The KGN cells were transfected with siTRIM28, and the intracellular SA β-Gal levels werre measured with a SA β-Gal staining kit (*n* = 3), magnification, 200×. (**E**) Western blot results of P16, P21, and γ-H2AX in KGN cells transfected with siTRIM28. (**F**) The MMP levels in KGN cells transfected with siTRIM28. (**G**) Intracellular ATP levels in KGN cells transfected with siTRIM28, scale bar: 100 μm. * *p* < 0.05, ** *p* < 0.01, *** *p* < 0.001, compared with the control-hGCs or NC group, Student’s *t*-test.

TRIM28 is a regulator of autophagy that inhibits autophagy by promoting the proteasomal degradation of AMPK [50]. A recent study reported that TRIM28 plays an important role in maintaining gonadal characteristics of the ovary and preventing the differentiation of GCs [32]; thus, we wondered whether it is involved in the excessive autophagy-mediated cellular senescence of GCs. First, we examined the expression of TRIM28 in the GCs of POI patients using Western blotting. The immunoblotting results showed that TRIM28 protein expression was relatively low in the GCs of POI patients compared with controls (Figure 4A). The results of immunofluorescence staining also showed a lower level of TRIM28 protein in GCs of ovaries with POI mice (Figure S1A). Meanwhile, we also constructed the linear regression and Pearson correlation between the relative expression levels of TRIM28 in the ovaries of POI mice and the serum levels of AMH, FSH, and E_2_ in the POI mice, and then found that the levels of TRIM28 were positively correlated with the levels of E_2_ and AMH, and negatively correlated with the levels of FSH (Figure S1B–D), suggesting that the expression levels of TRIM28 are associated with the progression of POI.

To clarify the role of TRIM28 in autophagy and cellular senescence in GCs, we knocked down the expression of TRIM28 using RNAi. Both siRNAs were effective in knocking down TRIM28, which resulted in the upregulation of the protein levels of the autophagy-related proteins LC3B-II and ATG5, and suppressed P62 accumulation (Figure 4B). We found that the reduction in TRIM28 protein levels resulted in elevated SA β-Gal activity (Figure 4C,D). Consistent with these results, the protein levels of cellular senescence markers P16, P21, and γ-H2AX were also significantly upregulated upon TRIM28 knockdown (Figure 4E). Moreover, the siTRIM28 group showed reduced MMP (Figure 4F) and ATP levels (Figure 4G) compared with the NC group. These results suggest that reduced TRIM28 expression promoted autophagy and further accelerated cellular senescence in GCs.

### 3.6. TRIM28 Attenuates OS-Induced Senescence-Associated Phenotypes by Inhibiting Autophagy in KGN Cells

**Figure 5:**
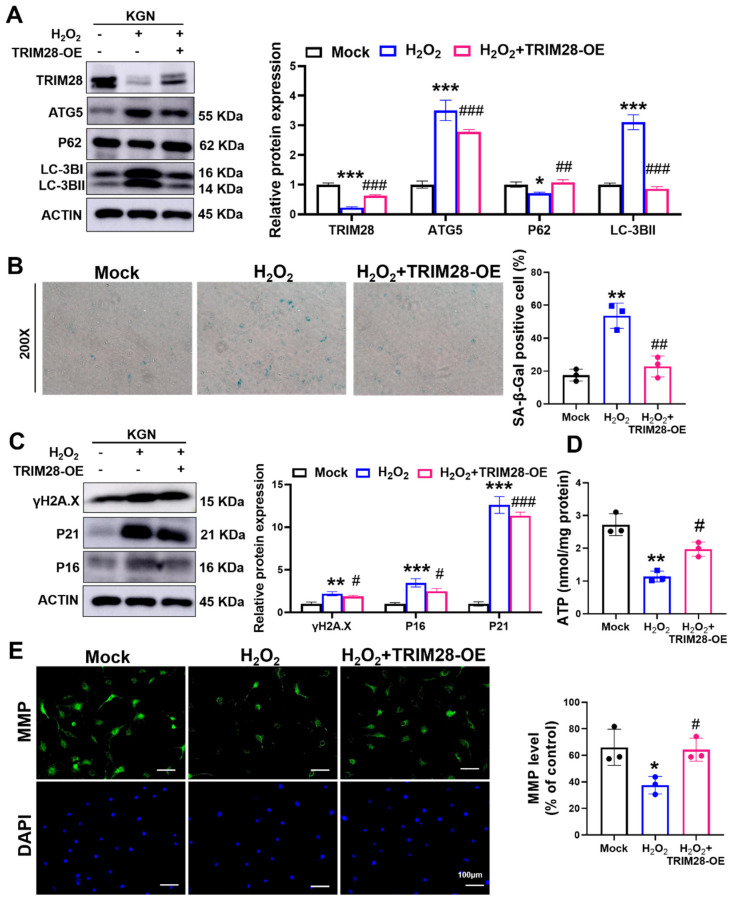
TRIM28 attenuates OS-induced senescence by suppressing autophagy in vitro. (**A**) The KGN cells were pre-infected with Mock or TRIM28-OE virus for 48 h, then treated with or without 100 μM H_2_O_2_ for 24 h, and the protein levels of TRIM28, LC3B, P62, and ATG5 were analyzed via Western blot. (**B**) After the treatment of KGN cells as described above, intracellular SA β-Gal levels were measured using an SA β-Gal staining kit (*n* = 3), magnification, 200×. (**C**) After the treatment of KGN cells as described above, the protein levels of P16, P21, and γ-H2AX were analyzed via Western blot. (**D**) After the treatment of KGN cells as described above, intracellular ATP levels were measured. (**E**) After the treatment of KGN cells as described above, the MMP levels were measured, scale bar: 100 μm. * *p* < 0.05, ** *p* < 0.01, *** *p* < 0.001, compared with the Mock group; ^#^ *p* < 0.05, ^##^ *p* < 0.01, ^###^ *p* < 0.001, compared with the H_2_O_2_-treated group, one-way ANOVA.

To clarify the role of TRIM28 in autophagy-dependent OS-induced cellular senescence, we pre-infected KGN cells for 48 h using an overexpressed TRIM28 lentiviral vector (TRIM28-OE). We found that the overexpression of TRIM28 reversed the H_2_O_2_-caused alterations in the protein levels of autophagy-associated proteins TRIM28, LC3B, ATG5, and P62 in KGN cells (Figure 5A), indicating an inhibitory effect of TRIM28 on OS-induced autophagy.

Next, we examined the senescence-related phenotypes and found that the overexpression of TRIM28 significantly alleviated the H_2_O_2_-induced elevation of SA β-Gal activity in KGN cells (Figure 5B) and reduced the expression levels of the senescence markers P16, P21, and γ-H2AX (Figure 5C). In addition, TRIM28-OE lentiviral pretreatment also rescued the OS-induced decrease in ATP (Figure 5D) and MMP levels (Figure 5E). The above results reveal that TRIM28 mediates the mechanism of cellular senescence caused by OS and that TRIM28 exhibits an anti-aging effect in KGN cells.

### 3.7. Overexpression of TRIM28 Improves Fertility in POI Mice

**Figure 6:**
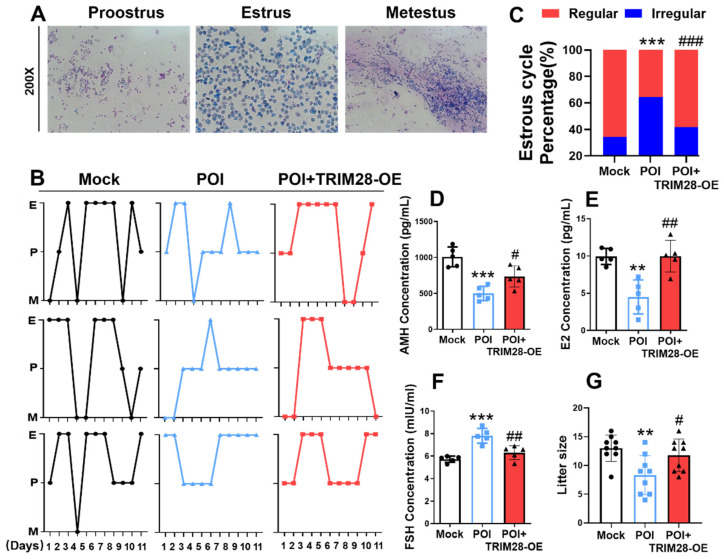
The effect of TRIM28 overexpression on reproductive function in mice. (**A**) Representative estrous cycles of mice in the Mock, POI, and POI + TRIM28-OE groups (*n* = 5), magnification, 200×. (**B**,**C**) Representative estrous cycles and the proportion of regular or irregular estrous cycles of mice in the Mock, POI, and POI + TRIM28-OE groups (*n* = 5). (**D**–**F**) The AMH (**D**), E_2_ (**E**), and FSH (**F**) level of mice in the Mock, POI, and POI + TRIM28-OE groups (*n* = 5). (**G**) The litter size of total mated mice (*n* = 9). ** *p* < 0.01, *** *p* < 0.001, compared with the Mock group; ^#^ *p* < 0.05, ^##^ *p* < 0.01, ^###^ *p* < 0.001, compared with the POI group, one-way ANOVA.

In vivo, the effects of TRIM28 on reproductive function in POI mice were evaluated by microinjecting TRIM28-OE lentivirus into mouse ovaries; the mice were mated after 7 days. We found that the estrous cycle of POI mice became irregular, whereas the overexpression of TRIM28 resulted in reduced estrous cycle irregularity in POI mice (Figure 6A–C). Mice in the POI group showed reduced levels of AMH and E_2_, and elevated levels of FSH in peripheral blood, while the levels of the relevant hormones were restored in the POI mice in the TRIM28-OE group (Figure 6D–F). Importantly, the overexpression of TRIM28 in the ovary resulted in a significant increase in the litter size in POI mice (Figure 6G). These results suggest that the overexpression of TRIM28 restores fertility in mice.

### 3.8. Overexpression of TRIM28 Attenuates Autophagy and Oxidative Stress, and Restores Ovarian Reserve In Vivo

**Figure 7:**
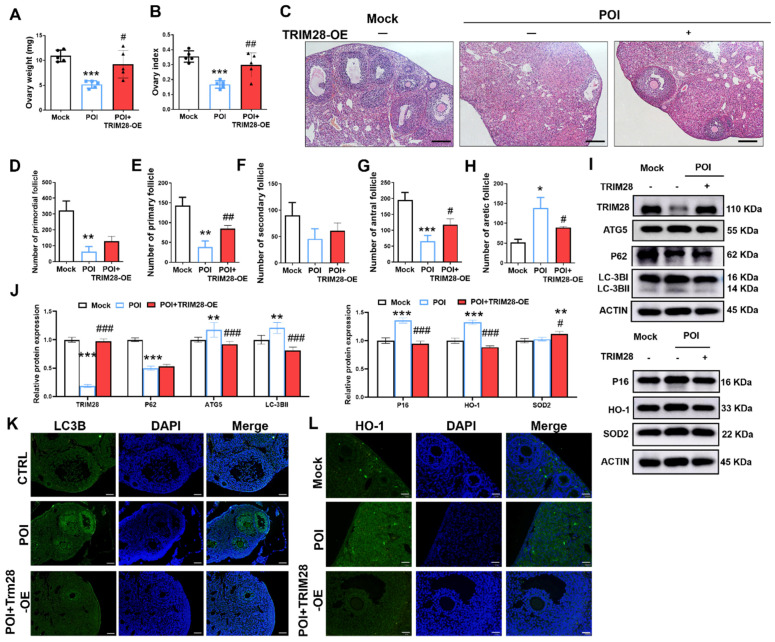
The effect of TRIM28 overexpression on the ovarian reserve. (**A**,**B**) Ovary weight and ovarian index of mice in the Mock, POI, and POI + TRIM28-OE groups (*n* = 5). (**C**) The representative H&E staining images of murine ovaries, magnification, scale bar: 50 μm. (**D**–**H**) Follicle counting results according to ovary serial sections (*n* ≥ 4). (**I**,**J**) The protein levels of TRIM28, LC3B, ATG5, P62, P16, P21, and γ-H2AX were analyzed via Western blot in the Mock, POI, and POI + TRIM28-OE groups. All experiments were repeated at least three times, and the results of representative experiments are shown. (**K**,**L**) Immunofluorescence staining of murine ovaries in the Mock, POI and POI + TRIM28-OE groups, scale bar: 20 μm. * *p* < 0.05, ** *p* < 0.01, *** *p* < 0.001, compared with the Mock group; *^#^ p* < 0.05, *^##^ p* < 0.01, *^###^ p* < 0.001, compared with the POI group, one-way ANOVA.

Next, we further assessed the effect of the overexpression of TRIM28 on ovarian function in POI mice. We found that the percentage ovary weight and index were both significantly increased in the POI + TRIM28-OE group compared to the POI group (Figure 7A,B). At 3 weeks of TRIM28-OE, the H&E staining revealed that the number of antral follicles (ANFs) and primary follicles (PFs) were significantly higher in the POI + TRIM28-OE group compared with the POI group. In addition, the number of atretic follicles (ATFs) was significantly decreased in the ovaries of POI + TRIM28-OE group compared with the POI group (Figure 7C–H), while the follicle proportion of primordial follicles (PMFs) and secondary follicles (SFs) showed no obvious differences between the POI group and POI + TRIM28-OE group (Figure 7C–H). These results suggested that the overexpression of TRIM28 reduces follicular atresia and promotes follicular development.

The Western blot results showed that TRIM28 overexpression significantly decreased the protein levels of ATG5, P62, and LC3B-II, and increased the level of P62, P16, HO-1, and SOD2 in the ovaries of mice (Figure 7I,J). Furthermore, the immunofluorescence showed that LC3B and HO-1 expression was reduced in GCs of POI mice in the POI + TRIM28-OE group compared with the POI group (Figure 7K,L). These data suggest that TRIM28 attenuates autophagy, OS, and senescence in GCs, thus restoring ovarian reserve in POI mice.

## 4. Discussion

**Figure 8:**
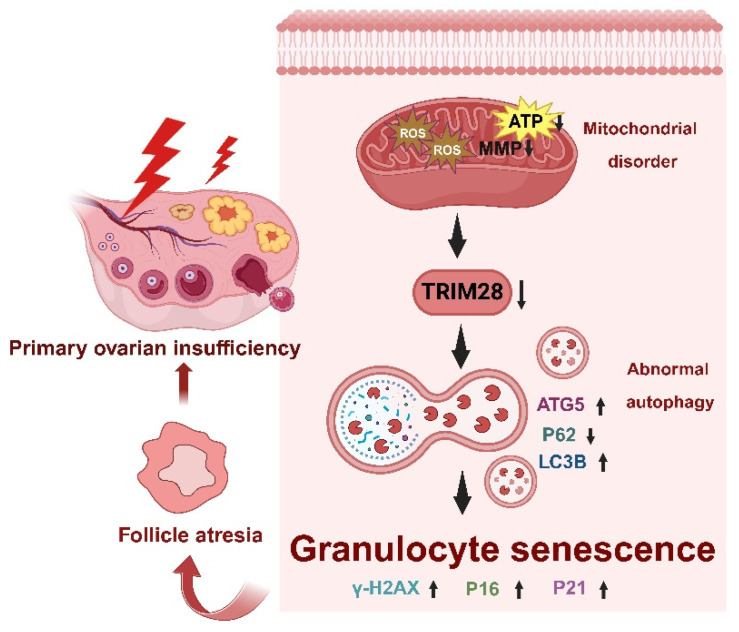
Schematic diagram of the molecular mechanism of the excessive OS mediated by TRIM28 which induces cellular senescence in bPOI-GCs.

Premature ovarian insufficiency (POI) is characterized by impaired ovarian function, resulting in reduced oocyte and follicle production, ultimately leading to infertility [51]. Oxidative stress (OS) plays a pivotal role in the pathogenesis of female reproductive disorders, triggering cell death pathways such as autophagy, apoptosis, and necrosis within ovarian follicles, thereby causing degenerative changes and POI [52]. Multiple studies have confirmed the presence of OS and autophagy in granulosa cells (GCs) of follicular fluid (FF) from bPOI patients [35,53,54]. In this study, the hGC line KGN and mice were employed to investigate the mechanism of OS-associated cellular senescence and to elucidate the role of TRIM28 as a critical mediator of POI pathogenesis in female reproduction. The knockdown of TRIM28 promoted autophagy in GCs and triggered cellular senescence, thereby accelerating POI. Both in vivo and in vitro experiments treated with TRIM28-OE lentivirus exhibited reduced ROS-induced oxidative damage to mitochondria, as well as a suppression of autophagy and a restoration of reproductive function, suggesting that OS-induced mitochondrial dysfunction and the downregulation of TRIM28 trigger cellular senescence via increased autophagy and further provoke the development of POI (Figure 8).

The pathogenic mechanism of POI is complex and poorly understood. The maturation of ovarian follicles is intricately linked to the support provided by GCs [55]. Previous studies have identified apoptosis and meiotic abnormalities in ovarian GCs as important causes of POI [56]. We have provided direct evidence that the abnormal aging of GCs is associated with POI. An increased activity of senescence-associated β-galactosidase (SA β-Gal) and elevated levels of senescence-related markers were found in the GCs of patients with bPOI. Furthermore, we observed a negative correlation between SA β-Gal activity and E_2_ and AMH levels, as well as the oocyte count, and a positive correlation with FSH levels.

OS is characterized by the excessive production of reactive oxygen species (ROS) or disorders in antioxidant regulation [4,5]. In bPOI-GCs, the level of ROS and the end product of lipid peroxidation, malondialdehyde (MDA), were significantly increased compared to normal GCs. However, excessive ROS production often correlates with mitochondrial dysfunction. Our study observed a substantial decrease in ATP levels and MMP in GCs of bPOI patients, which is consistent with our projections. Mitochondria are crucial organelles that regulate cellular metabolism and signal transduction while maintaining vital cell functions [43,44]. Excess ROS is catalyzed by mitochondrial SOD2 to form H_2_O_2_, which is then decomposed into non-toxic water by catalase [57]. H_2_O_2_ was used to treat the GC line KGN to induce senescence, and the addition of the antioxidant N-acetylcysteine (NAC) was observed to attenuate cellular senescence, further confirming the OS involved in the GC senescence process.

The activation of autophagy by OS has been extensively documented [58,59,60,61]. Under physiological conditions, autophagy plays a pivotal role in various processes such as development, aging, and immunity [62]. In this study, we observed an increase in the protein levels of senescence markers P16, P21, and γ-H2AX and an upregulation in the protein expression levels of autophagy-associated proteins LC3B-II and ATG5, as well as an increase in the level of P62 in H_2_O_2_-treated GCs. Importantly, treatment with the autophagy inhibitor CQ significantly alleviated GC aging, providing abundant evidence that ROS is involved in autophagy-dependent mechanisms that induce GC senescence.

Recently, the role of TRIM28 in maintaining embryo development and embryonic stem cell (ESC) multifunctionality has received increasing attention [63,64,65], yet the underlying mechanism remains elusive. In this study, we focused on the role of TRIM28 in regulating autophagy and ROS in hGCs. To achieve this, we utilized si-RNA to inhibit TRIM28 expression in KGN cells. Following siTRIM28 treatment, we observed an upregulation of autophagy-specific genes LC3B-II and ATG5, along with a decrease in P62 levels, and a significant increase in the protein levels of senescence markers P16, P21, and γ-H2AX. The above suggests that the low expression of TRIM28 can contribute to the autophagic and senescent phenotypes of KGN cells, which are consistent with the GCs of bPOI patients. In addition, we observed that the levels of TRIM28 in the ovaries of POI mice were positively correlated with the levels of E_2_ and AMH and negatively correlated with the levels of FSH (Figure S1B–D). And the expression of POI-causing genes such as FOXL2, FSHR, AMH, and CYP19A1 was significantly decreased in the siTRIM28-treated group (Figure S2). It is indicated that TRIM28 is associated with the onset of POI.

To further ensure the correlation between TRIM28 and ovarian function, we used the TRIM28-overexpression lentivirus and found that the treatment with TRIM28-OE lentivirus produced a significant recovery effect on H_2_O_2_-induced KGN cells. More importantly, upon injection of the TRIM28-OE lentivirus into the ovaries of POI mice, the follicular development, hormone secretion, and estrous cycles were markedly improved, accompanied by an improved fertility rate, further supporting our results that knocking down TRIM28 in GCs affects normal cell function. As we know, the level of AMH is a sensitivity indicator of ovarian dysfunction, which can be used as a marker for reflecting ovarian reserve, while AMH was mainly expressed in GCs of ANFs and 4 mm-diameter small antral follicles and not in ATFs [66]. In this study, we detected that serum AMH levels markedly increased in the POI + TRIM28-OE mice compared with the POI mice. Relevant to this, notable alterations in the number of antral follicles (ANFs) were also observed in the POI + TRIM28-OE mice compared with the POI mice. Additionally, the level of FSH and E_2_ showed that there were significant changes in POI + TRIM28-OE mice and POI mice, which reflects that the hypothalamus vertical excitation element has a noteworthy difference between POI + TRIM28-OE mice and POI mice. These results also proved once again that the TRIM28 plays a crucial role in the treatment of POI. Furthermore, the expression levels of FOXL2, FSHR, AMH, and CYP19A1 genes were significantly elevated in the TRIM28-OE group compared to those in the POI group (Figure S3). Our results provide further evidence that TRIM28 ameliorates the senescence of GCs associated with POI by inhibiting autophagy in vitro and in vivo.

Although we believe that TRIM28 plays a pivotal role in the regulation of GC aging through autophagy, further exploration of a more precise and comprehensive signaling pathway is warranted for future studies. In subsequent studies, we aim to investigate the effects of TRIM28 regulatory pathways on GC function in patients with bPOI at different stages. In summary, our research has contributed to an enhanced understanding of the mechanisms underlying follicular cell depletion and has provided novel avenues for intervening in ovarian aging, thereby offering potential therapeutic strategies for POI.

## Data Availability

Data are contained within the article and Supplementary Materials.

## Supplementary Materials

The following supporting information can be downloaded at: https://www.mdpi.com/article/10.3390/antiox13030308/s1, Table S1. The list of human follicular fluid samples; Figure S1. The level of TRIM28 in the ovaries of POI mice. (A) Immunofluorescence staining of murine ovaries in Ctrl and POI groups, scale bar: 20 μm. (B–D) Scatter diagram showing linear regression and significant Pearson correlation between the relative expression level of TRIM28 and level of AMH (B), FSH (C), and E_2_ (D) in POI on quantitative results of immunofluorescence staining (*n* = 10); Figure S2. The RT-qPCR of key genes during the occurrence of premature ovarian failure in KGN cells transfected with siTRIM28, (*n* = 3). ** *p* < 0.01, *** *p* < 0.001, compared with the NC group, Student’s *t*-test; Figure S3. The RT-qPCR of key genes during the occurrence of premature ovarian failure in Mock, POI, and POI + TRIM28-OE groups, (*n* = 3). *** *p* < 0.001, compared with the Mock group; ## *p* < 0.01, ### *p* < 0.001, compared with the POI group, one-way ANOVA.
