# Supplementary material for: TRIM28-Mediated Excessive Oxidative Stress Induces Cellular Senescence in Granulosa Cells and Contributes to Premature Ovarian Insufficiency In Vitro and In Vivo
Source: Antioxidants (Basel). 2024 Mar 1;13(3):308. doi: 10.3390/antiox13030308 (PMC10967454; doi:10.3390/antiox13030308)
Supplement: Supplementary file 1 [file antioxidants-13-00308-s001.zip › antioxidants-2824675-supplementary.pdf]

Supplementary material:

TRIM28-Mediated Excessive Oxidative Stress Induces Cellular Senescence in Granulosa Cells and Contributes to Premature Ovarian Insufficiency In Vitro and In Vivo

Chong Zhou 1,2,†, Dandan Li 2,3,†, Jinxia He 4, Tao Luo 1,2,5, Yiting Liu 1,2, Yue Xue 1,2, Jian Huang 2, Liping Zheng 2,3,6,7,* and Jia Li 1,2,*

1 School of Basic Medical Sciences, Jiangxi Medical College, Nanchang University, Nanchang 330031, China; 356400210004@email.ncu.edu.cn (C.Z.); luotao@ncu.edu.cn (T.L.); 406400230104@email.ncu.edu.cn (Y.L.); 356400220010@email.ncu.edu.cn (Y.X.)

2 Key Laboratory of Reproductive Physiology and Pathology of Jiangxi Province, Jiangxi Medical College, Nanchang University, Nanchang 330031, China; 4202120020@email.ncu.edu.cn (D.L.); jianhuang@ncu.edu.cn (J.H.)

3 HuanKui College, Nanchang University, Nanchang 330031, China

4 Reproductive Medical Center, Jiangxi Maternal and Child Health Hospital, Affiliated Maternal and Child Health Hospital of Nanchang University, Nanchang 330006, China; 401428818004@email.ncu.edu.cn

5 Institute of Life Science, Nanchang University, Nanchang 330031, China

6 School of Public Health, Jiangxi Medical College, Nanchang University, Nanchang 330006, China

7 Jiangxi Provincial Key Laboratory of Preventive Medicine, Jiangxi Medical College, Nanchang University, Nanchang 330006, China

* Correspondence: zhengliping@ncu.edu.cn (L.Z.); lijia4199@ncu.edu.cn (J.L.)

† These authors contributed equally to this work.

Table. S1 The list of human follicular fluid samples

	NO.	Age	FSH	E2	LH	AMH	Oocyte
bPOI	1	31	12	10.7	4.33	0.392	3
	2	33	10.8	34.8	7.23	1.38	7
	3	33	11.18	25.1	4.33	1.12	5
	4	31	10.2	34.3	3.36	2.59	5
	5	33	11.1	57.1	10.3	2.15	3
	6	36	16.7	18	5.06	0.41	3
	7	34	10.6	23.9	4.01	0.05	3
	8	44	29.1	20.9	10.7	0.213	2
	9	30	17.5	5.01	1.15	0.257	4
	10	38	10.9	43	7.89	0.57	6
	11	43	10.6	22.7	4.63	2.04	10
	12	35	13.4	31.4	7.38	1.46	8
	13	45	14.98	32.728	9.23	0.08	1
	14	42	10.6	19.78	2.29	0.28	3
	15	37	10.2	26.45	6.97	0.19	1
Ctrl	1	29	3.94	193	6.47	6.83	14
	2	32	6.34	78.6	5.46	4.61	14
	3	32	3.11	111	5.82	4.23	11
	4	38	7.21	132	4.07	3.84	23
	5	31	5.98	76.5	1.92	3.36	16
	6	34	4.95	81.2	5.05	3.8	17
	7	35	4.22	128	4.97	5.04	15
	8	26	6.19	243	33.7	3.14	20
	9	31	2.92	144	4.49	5.17	13
	10	29	5.83	88.4	12.9	5.05	28
	11	26	6.57	44.9	3.17	6.46	13
	12	35	5.66	179	7.13	1.93	5
	13	30	4.42	344	2.73	4.06	19
	14	24	1.61	204.27	2.7	2.93	19
	15	33	4.59	66.84	7.59	5.8	8

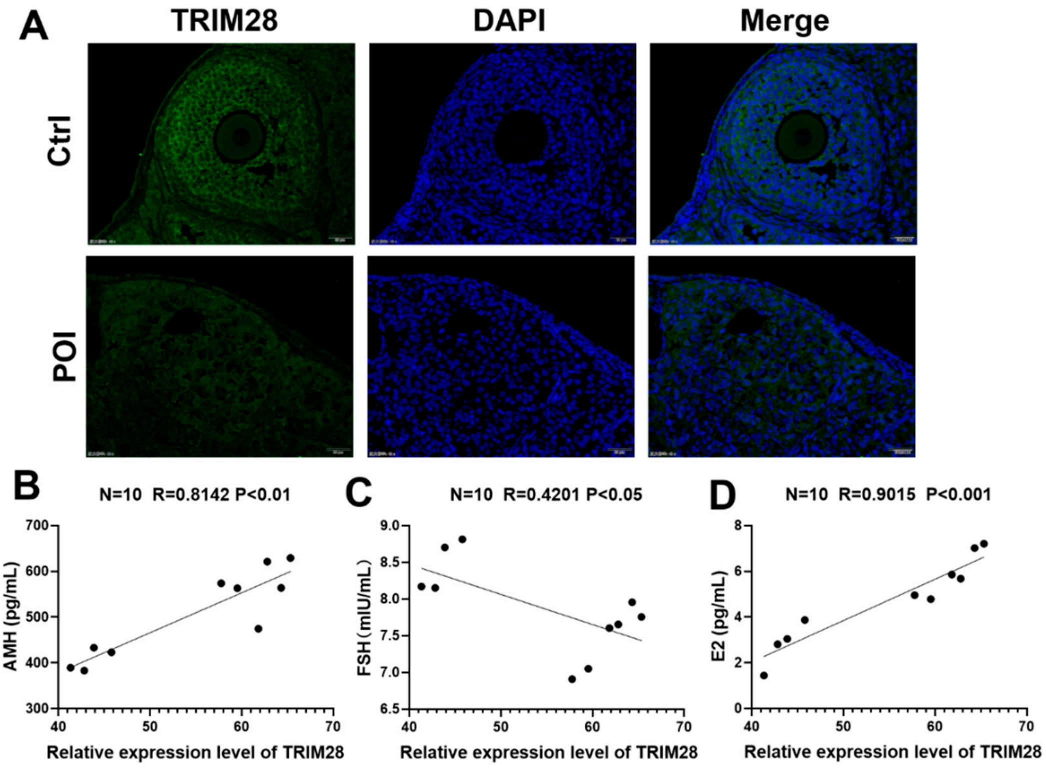

Figure S1. The level of TRIM28 in the ovaries of POI mice. (A) Immunofluorescence staining of murine ovaries in Ctrl and POI groups, scale bar: 20 μm. (B–D) Scatter diagram showing linear regression and significant Pearson correlation between the relative expression level of TRIM28 and level of AMH (B) , FSH (C) and E_2 (D) in POI on quantitative results of Immunofluorescence staining (n = 10).

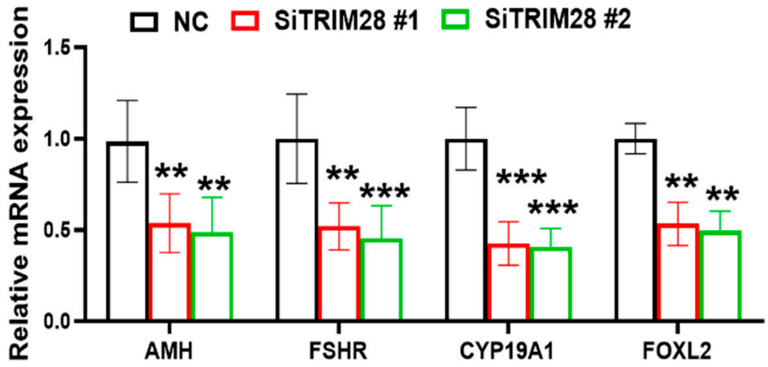

Figure S2. The RT-qPCR of key genes during the occurrence of premature ovarian failure in KGN cells transfected with siTRIM28, (n = 3). **p < 0.01, ***p < 0.001, compared with the NC group, Student's t–test.

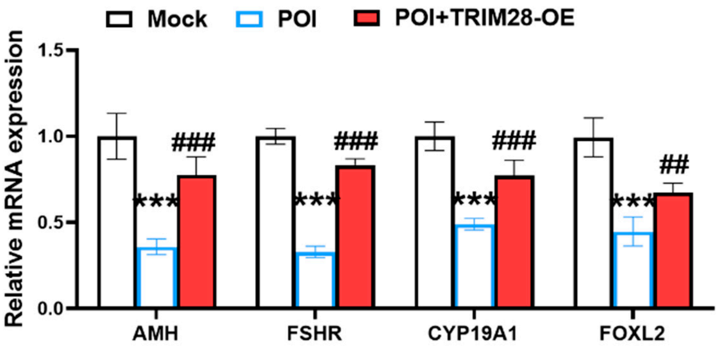

Figure S3. The RT-qPCR of key genes during the occurrence of premature ovarian failure in Mock, POI, and POI + TRIM28–OE groups, (n = 3). ***p < 0.001, compared with the Mock group; ##p < 0.01, ###p < 0.001, compared with the POI group, One-way ANOVA.
